# Supplementary material for: In Tandem Control of La-Doping and CuO-Heterojunction on SrTiO3 Perovskite by Double-Nozzle Flame Spray Pyrolysis: Selective H2 vs. CH4 Photocatalytic Production from H2O/CH3OH
Source: Nanomaterials (Basel). 2023 Jan 25;13(3):482. doi: 10.3390/nano13030482 (PMC9920848; doi:10.3390/nano13030482)
Supplement: Supplementary file 1 [file nanomaterials-13-00482-s001.zip › nanomaterials-2164592-supplementary.pdf]

Supplementary Materials

In Tandem Control of La-Doping and CuO-Heterojunction on $SrTiO_3$ Perovskite by Double-Nozzle Flame Spray Pyrolysis: Selective H_2 *vs*. CH_4 Photocatalytic Production from H_2O/CH_3OH

Pavlos Psathas [1], Areti Zindrou[1], Christina Papachristodoulou[1], Nikos Boukos[2] and Yiannis Deligiannakis[1,*]

[1] Department of Physics, University of Ioannina, Ioannina 45110, Greece
[2] Institute of Nanoscience and Nanotechnology (INN), NCSR Demokritos, Athens 15310, Greece
* Correspondence: ideligia@uoi.gr; Tel.: +302-651-008-662

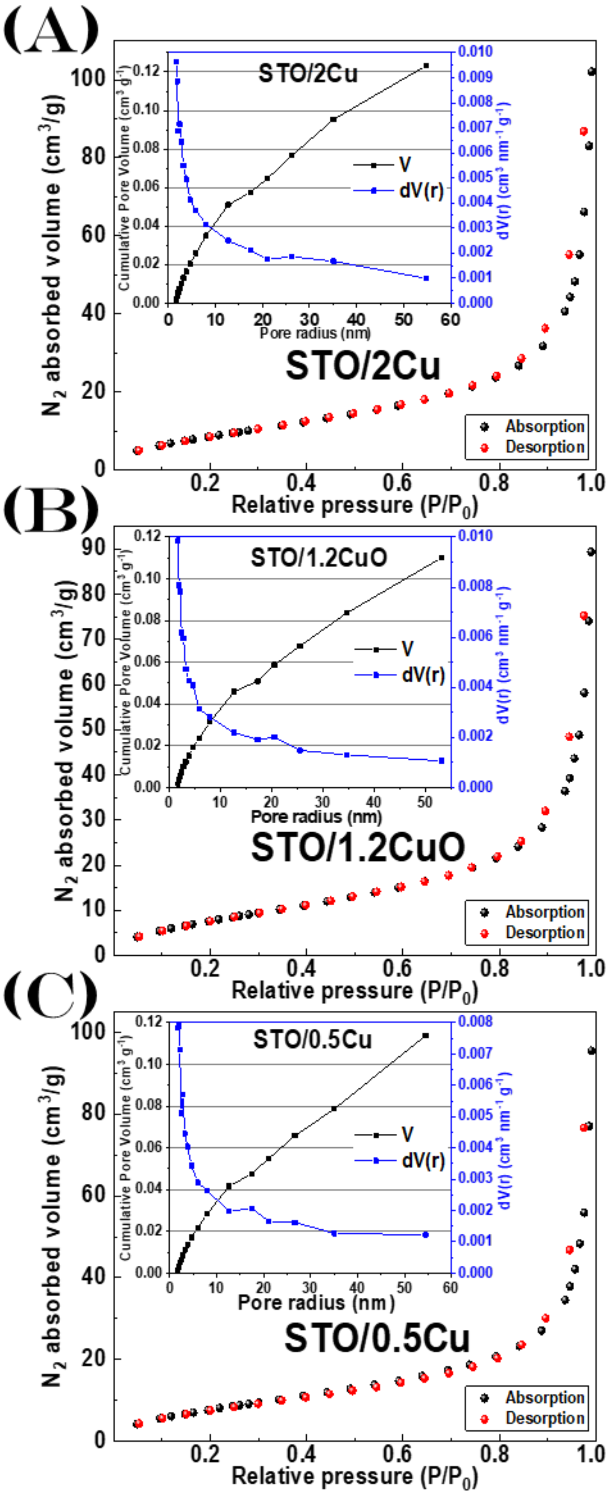

Figure S1. (A-C) N_2 adsorption-desorption isotherms of 2%, 1.2%, and 0.5% CuO materials, Inset: Pore size distribution plot using the BJH method.

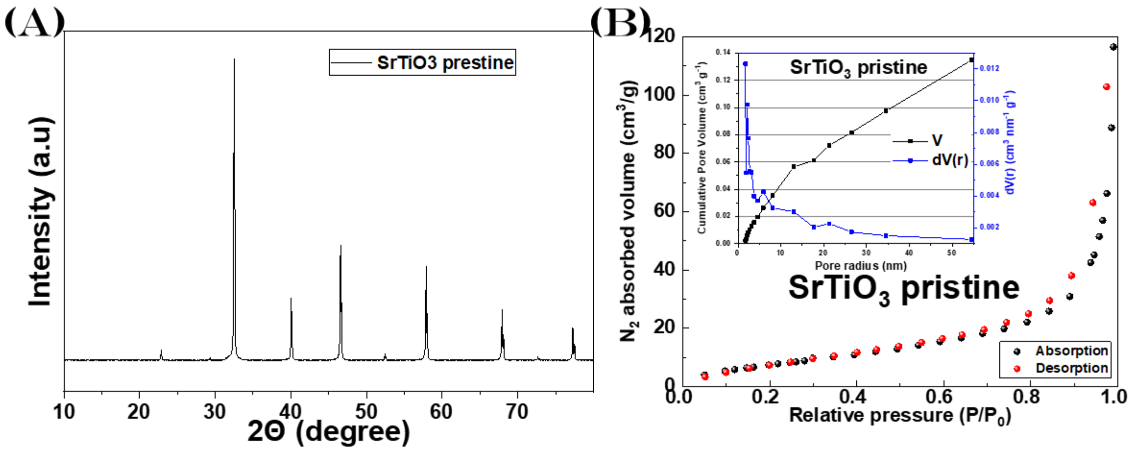

Figure S2. (A) XRD of the pristine $SrTiO_3$, (B) N_2 adsorption-desorption isotherms of pristine $SrTiO_3$, Inset: Pore size distribution plot using the BJH method.

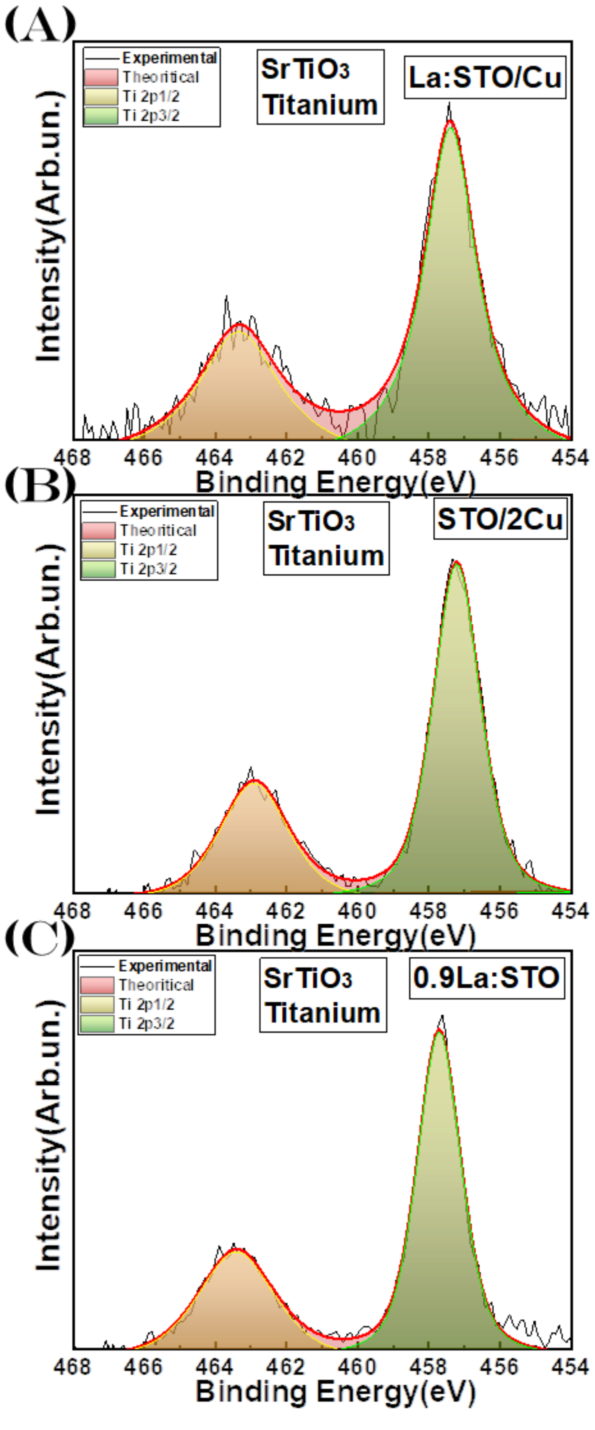

Figure S3. XPS data for the nanomaterials $SrTiO_3$ for the (A) 0.25%La + 0.5%Cu, (B) 2%Cu, (C) 0.9%La for the Ti binding energies.

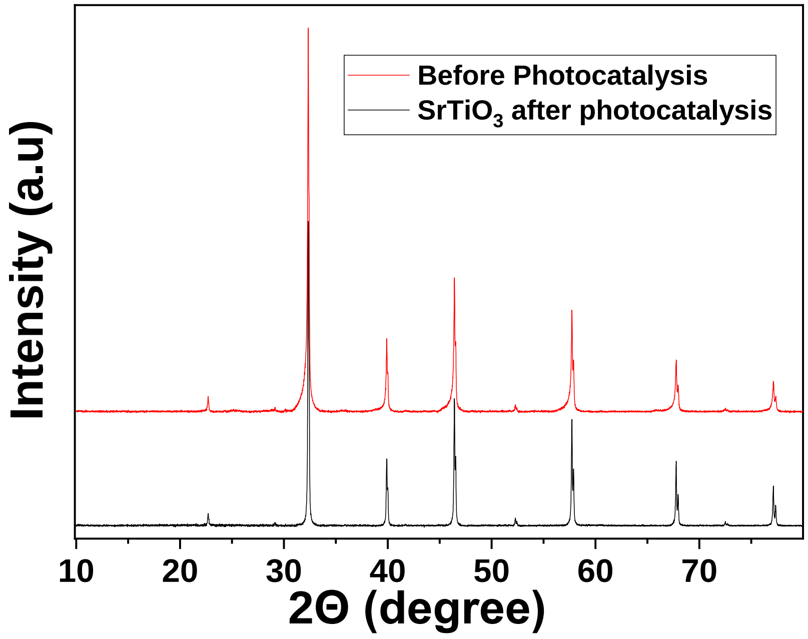

Figure S4. XRD data for the before and after photocatalysis.
